# Use of high- and low-intensity lasers in the treatment of dentin hypersensitivity: A literature review

**DOI:** 10.4317/jced.57783

**Published:** 2021-04-01

**Authors:** Thamyres-Maria-Silva Simões, Kamila-Cibele-Bezerra Melo, José-de Alencar Fernandes-Neto, Ana-Luzia-Araújo Batista, Maria-das Graças-Barbosa da Silva, Alieny-Cristina-Duarte Ferreira, Juliane-Alves de Sousa, Maria-Helena-Chaves-de Vasconcelos Catão

**Affiliations:** 1 PhD student, Postgraduate Program in Dentistry, State University of Paraíba (UEPB), Campina Grande, Paraíba, Brazil; 2 Graduate in Dentistry, State University of Paraiba (UEPB), Campina Grande, Paraíba, Brazil; 3 Master’s student, Postgraduate Program in Dentistry, State University of Paraíba (UEPB), Campina Grande, Paraíba, Brazil; 4 Dentistry student, State University of Paraiba (UEPB), Campina Grande, Paraíba, Brazil; 5 PhD Professor, Postgraduate Program in Dentistry, State University of Paraíba (UEPB), Campina Grande, Paraiba, Brazil

## Abstract

**Background:**

Dentin hypersensitivity (DH) is defined as an exaggerated sensitivity of vital dentin exposed to thermal, chemical and tactile stimuli. This study aimed to evaluate, through a literature review, the applicability of high- and low-intensity lasers in the treatment of DH for the past 10 years, as well as its therapeutic potential.

**Material and Methods:**

The electronic databases MEDLINE/PubMed and LILACS were searched using the descriptors (“Dentin Sensitivity” OR “Dentin Hypersensitivity”) AND (“Low-Level Therapy” OR Laser), for articles published between 2010 and 2020. Only randomized clinical trials with full-text and full case resolution were included.

**Results:**

We found 187 articles in total, among which 61 were pre-selected and 10 included in this literature review.

**Conclusions:**

Considering the found results and their possible limitations, high- and low-intensity lasers, associated or not with other therapies, have demonstrated beneficial effects in the treatment of DH, being considered a promising, safe, easy, and effective field of research, reducing pain sensitivity and preserving pulp vitality.

** Key words:**Dentin sensitivity, dentistry, laser.

## Introduction

Dentin hypersensitivity (DH) is a sharp, severe, and sudden pain response, dissociated from any other form of dental pathology, arising from dentin exposure to thermal, evaporative, tactile, osmotic or chemical stimuli (1,2).

The prevalence of DH is highly heterogeneous, estimated at around 3.8% to 85% (3-6), being a common occurrence in health services. Age and sex are directly related to its high prevalence, with young individuals, between 18 and 44 years old, being female, and with gingival recession, the most affected by DH (6,7).

The diagnosis and treatment of DH are complex, due to their multifactorial nature and, for this reason, hypersensitive teeth should be carefully examined regarding pulp and gingival health (8,9). Dentin exposure often results from cementum removal from the cervical region, gingival recession, and enamel removal associated with different types of tooth wear (10). Histologically, hypersensitive dentin presents enlarged dentinal tubules and in greater number per surface area than dentinal without sensitivity, for this reason, DH causing discomfort and, in more severe situations, interfering with the individual’s quality of life (11).

Researchers have proposed several theories to explain the mechanism of DH. Brannstrom’s hydrodynamic theory is currently the most widely accepted by the literature. It claims that dentinal tubules are filled with a fluid that, under stimulus, moves and stimulates the retraction or distension of odontoblast processes, reaching nerve endings at the dentin-pulp interface and generating pain (12,13).

The Lights Amplification by Stimulated Emission of Radiation (Lasers) have been proposed as an alternative for treating DH and has become an area of interest for research in recent decades. Desensitization seems to depend mostly on the type of laser therapy adopted, high- or low-intensity (14).

High-intensity lasers operate on the obliteration of dentinal tubules by the direct irradiation of the exposed dentin after dentinal surface recrystallization. Such morphological change forms a layer on the target tissue, that can promote sealing up to 4.0μm deep within dentinal tubules, eliminating pain sensitivity for an extended period (15,16).

The low-intensity lasers in the treatment of DH, has been gaining prominence in the literature due to the proof of its clinical use, may achieve satisfactory results after the first session and continuous analgesia of the irradiated region, for an extended period, even after treatment ends (17).

At first, low-intensity lasers may increase excitability threshold of nerve endings, inducing analgesia; it may also play a role in maintaining resting membrane potential in the nociceptive receptor. In the long term, they increase metabolic activity of odontoblasts, forming restorative dentin and obliterating dentin canaliculi (18).

Many aspects of laser use remain controversial, such as the best type of laser, parameters used, exposure time, and number of treatment sessions. Such aspects evoke the need for further clinical studies to determine a correct and effective protocol for the treatment of DH.

Thus, this study aimed to evaluate, through a literature review, the applicability of high- and low-intensity lasers in the treatment of DH for the past 10 years, as well as its therapeutic potential.

## Material and Methods

The study is characterized as a literature review, carried out in the electronic databases MEDLINE / PubMed and LILACS, between April and May 2020, using the keywords (“Dentin Sensitivity” OR “Dentin Hypersensitivity”) AND (“Low-Level Light Therapy” OR Laser) in association.

Scientific papers found in data collection underwent an initial screening, considering titles and abstracts of articles published between 2010 and 2020. The pre-selected articles were evaluated regarding methodological detail of the research and consistency of the results presented by the authors. Only randomized clinical trials with full-text and full case resolution were included, as this type of study is considered the gold standard for guiding clinical practice. Articles published before 2010 and focused on themes other than laser use in the treatment of DH were excluded.

## Results

**Figure 1:**
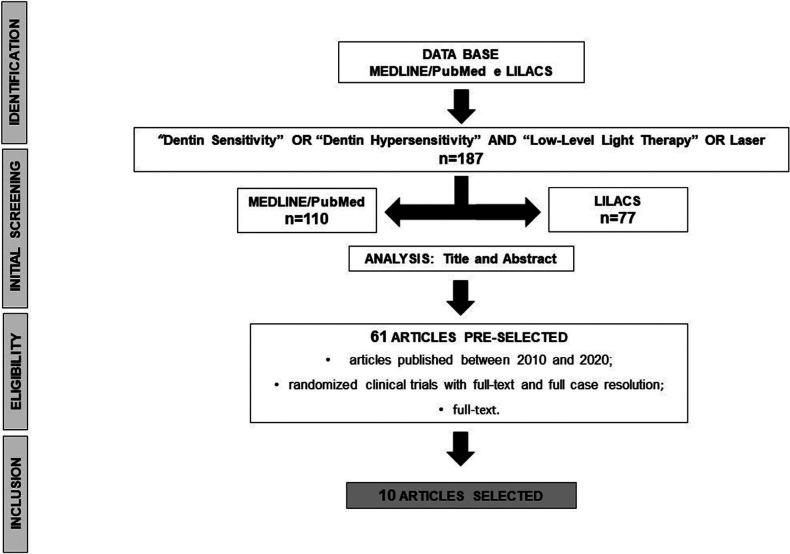
Flowchart showing the research strategy and articles selected for review.

We found 187 articles using the mentioned keywords. After analyzing the titles and abstracts, 61 articles were pre-selected and read in full, excluding studies in conflict with the established inclusion criteria. At last, 10 articles were selected for the present review (Fig. 1).

The characterization of the studies regarding the objectives, lasers used and their conclusions is shown in Table 1,  Table 1 cont.

**Table 1:**
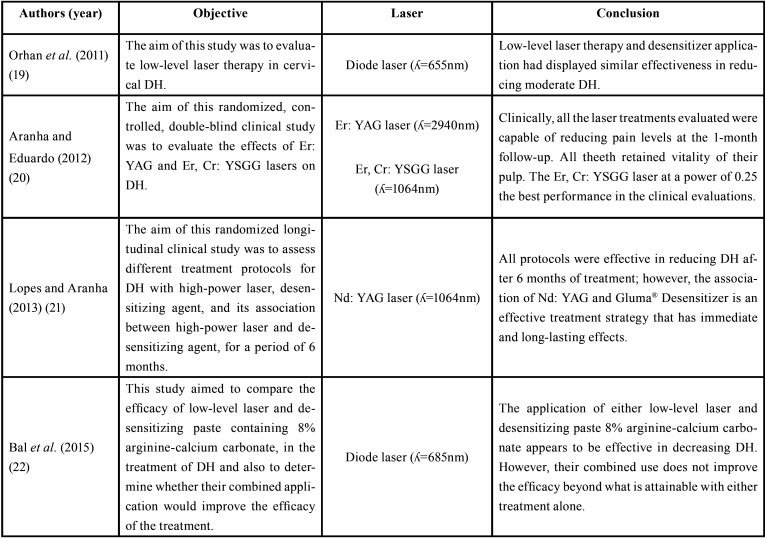
Selected studies according to the inclusion and exclusion criteria established by this review.

**Table 1 cont.**
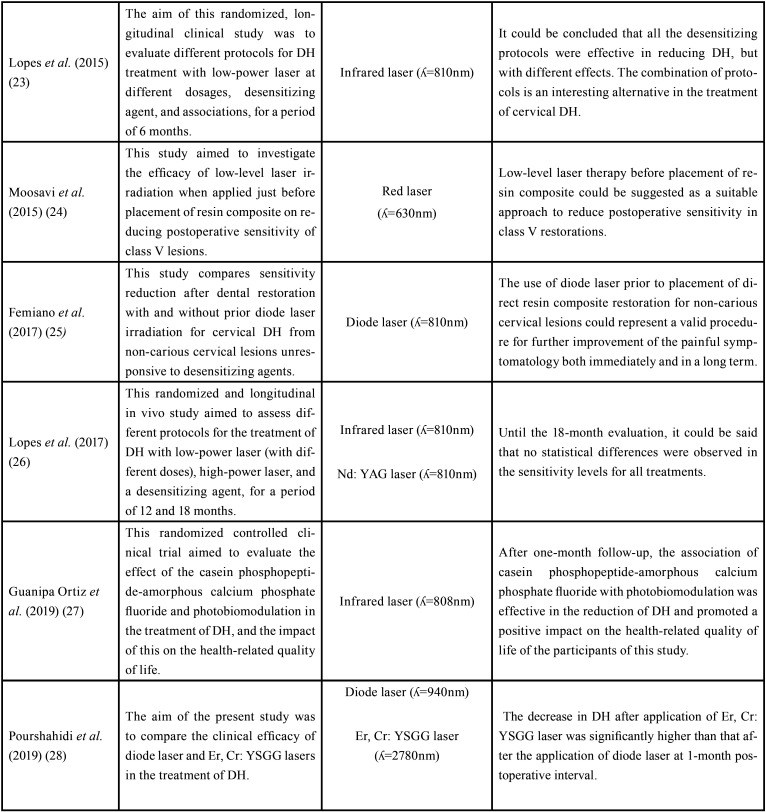
Selected studies according to the inclusion and exclusion criteria established by this review.

## Discussion

Treating DH consists of reducing fluid movement inside dentinal canaliculi, narrowing or occluding open tubules, which it blocks the transmission of nerve stimuli to odontoblasts, inhibiting pain (29). Several therapies have been proposed over the years (30-32), however, none has shown a long-term efficiency in managing pain symptoms caused by DH (33,34).

Lasers have been the focus of several research in Dentistry (35,36) and have shown to be a promising alternative to the treatment of DH (25,27,28). It should be considered that there is a diversity of protocols adopted in these studies, which demonstrates the complexity of DH and makes the comparison of results difficult (Table 1, 1 cont.).

For the treatment of DH, high-intensity lasers can be used, which promotes dentin sealing or alters tubular content by coagulation, protein precipitation, or the creation of insoluble calcium complexes (37,38), and low-intensity lasers promotes, at cellular level, analgesic, anti-inflammatory, and biostimulating effects (14).

Neodymium: yttrium-aluminum-garnet (Nd: YAG) laser was the first to be described in the literature (39) and, among high-intensity lasers, it has the greatest scientific and clinical prominence in relieving pain caused by DH, due to its ability to obliterate dentinal tubules by a process called fusion and resolidification, causing neither pulp damage nor cracks in the teeth. Lopes and Aranha (21) observed the benefits of Nd: YAG laser in treating DH, with immediate and lasting results, enhanced by its association with a desensitizing agent.

Despite such evidence, Lopes *et al.* (26) observed no statistical differences in pain sensitivity levels when comparing Nd: YAG laser to a low-intensity laser, in high and low doses, and a desensitizing agent. Possibly, occlusion may not have occurred in all dentinal tubules after Nd: YAG laser irradiation, considering the protocol adopted in this study, which would justify some patients’ pain.

Erbium lasers have likewise been used for treating DH. In a study conducted by Aranha and Eduardo (20), the use of high-intensity lasers for treating DH caused by non-carious cervical lesions presented promising results, reducing pain sensitivity and preserving pulp vitality. In this study, Erbium chromium: yttrium-scandium-gallium-garnet (Er, Cr: YSGG) laser had better clinical results than Erbium: yttrium-aluminum-garnet (Er: YAG) laser.

Similar results were reported by Pourshahidi *et al.* (28), when comparing low-intensity diode laser and Er, Cr: YSGG laser, significantly superior in reducing DH, after one month. Er, Cr: YSGG may attain better results, considering its ability in melting peritubular dentin, whereas diode laser is less effective in dentinal tubules occlusion (40).

Low-intensity lasers have gained great popularity among clinicians, and their immediate effect on pain relief in DH, depends mainly on the stimulation of pulp tissue nerve cells, interfering with the polarity of cell membranes and blocking nerve stimulation (33). Thus, as noticed by Moosavi *et al.* (24) and Femiano *et al.* (25), analgesic effects promoted by low-intensity lasers after class V restorations and non-carious cervical lesions, may be associated with suppression of nerve transmission at the pulp-dentin interface.

Besides the individual effects of laser, its use in combination has demonstrated additive or synergistic effects in the treatment of DH. Laser irradiation, when used in combination, is usually applied after the use of topical agents, ensuring a better coverage of dentin channels (21). Orhan *et al.* (19), Lopes *et al.* (23) and Guanipa Ortiz *et al.* (27) observed such synergism in relieving pain caused by DH and improving the quality of life of studied individuals. These findings suggest the potentiating effects of laser radiation when combined with a desensitizing agent.

However, the benefits of this association, contrast with the results found by Bal *et al.* (22), when combining low-intensity diode laser and desensitizing paste containing arginine-calcium carbonate. These results, perhaps, are related to the mechanisms of action of each treatment.

## Conclusions

Considering the found results and their possible limitations, high- and low-intensity lasers, associated or not with other therapies, have demonstrated beneficial effects in the treatment of DH, being considered a promising, safe, easy, and effective field of research, reducing pain sensitivity and preserving pulp vitality. Nevertheless, further studies are needed to develop a definitive protocol, allowing a better comparison of results available in the literature, and understanding of the exact mechanism of action of lasers in DH.
